# Temperament assessment of black (*Diceros bicornis*) and white (*Ceratotherium simum*) rhinos using keeper temperament surveys and novel object tests

**DOI:** 10.1371/journal.pone.0356484

**Published:** 2026-09-16

**Authors:** Kelly Chimpén Mac Leod, Marieke K. Jones, Lara C. Metrione, Elizabeth W. Freeman

**Affiliations:** 1 Environmental Science and Policy Department, George Mason University, Fairfax, Virginia, United States of America; 2 Department of Biology, George Mason University, Fairfax, Virginia, United States of America; 3 South‐East Zoo Alliance for Reproduction & Conservation, Yulee, Florida, United States of America; 4 School of Integrative Studies, George Mason University, Fairfax, Virginia, United States of America; Narula Institute of Technology, INDIA

## Abstract

Ex situ conservation of African rhinoceros is essential for their preservation, and characterization of temperament could be useful for managing their wellbeing. Here, the temperaments of 49 black (*Diceros bicornis*, BR) and 125 white (*Ceratotherium simum*, WR) rhinos in North American institutions were assessed using keeper temperament surveys. Median keeper ratings on 12 behaviors were clustered by PCAs into temperament profiles related to bold/fearful, active/inactive and anthrophilic/-phobic traits for BR and WR, and reactive/stoic traits for WR. Novel object (rope basket) tests were conducted on 32 BR and 66 WR to measure individual temperaments. Proportion of time active and stimulated, frequency of interactions, and latency to approach the basket were analyzed. Statistical models were used to determine associations of demographic and environmental factors with the expression of temperament. Spearman’s correlations were used to assess the relationship and validity between temperament assessments. The survey temperament profiles indicated that males of both species were more active than females, and activity declined with age in WR (*p* < 0.05). Age, temperature and enclosure size were significantly associated with behaviors in BR and WR during the novel object test, whereas sex, calf presence and social housing additionally had significant associations with WR behaviors. Younger BR and WR and individually-housed WR were more likely to engage in novel situations (*p* < 0.05). Personality traits of active/inactive, anthrophilic/-phobic, bold/fearful, exploratory/avoidant, and sociable/aggressive with conspecifics were correlated in both assessments (*p* < 0.05) reinforcing the reliability of temperament surveys and novel object tests for assessing rhino temperament. These assessments measured rhinos’ tendency towards fearfulness and exploration. Understanding temperament could aid managers making decisions related to training, housing, or other aspects to minimize aversive experiences. Consideration of a rhino’s natural activity and comfort with humans could enable tailoring expectations for daily behaviors, interactions with enrichment, conspecifics, and people to promote positive mental states and wellbeing.

## Introduction

African black (*Diceros bicornis*) and white (*Ceratotherium simum*) rhinos were brought to the brink of extinction due to poaching for their horns, with black rhinos decreasing by 98% to ~2,400 individuals in 1995 and white rhinos falling to only ~20–50 individuals in 1890 [1,2]. Currently, the black rhino population is estimated at ~3,142 individuals and listed by the International Union for Conservation of Nature (IUCN) Red List as critically endangered with an increasing trend [1,3]. The white rhino population is estimated at ~10,080 individuals and listed by the IUCN as near threatened with a decreasing trend [2,3]. Due to the risks rhinos face in the wild, ex situ conservation efforts play a crucial role in the preservation of these species. To maintain a self-sustaining ex situ rhino population, zoos and other rhino-holding institutions need to assess individual management, compatibility and health to enhance breeding programs and positive wellbeing [4–6].

One way to quantify and understand the wellbeing of rhinos is through the Five Domains Model, which focuses upon behavioral interactions, nutrition, environment, and health [6–8]. Rhino negative or positive responses to situational experiences from the first four domains determine the fifth domain, their mental state [7,8]. An individual’s temperament provides insight into situational interactions with conspecifics, humans, and its environment, and facilitates quantifying its mental state. Thus, assessing an individual’s temperament is essential for monitoring wellbeing.

Temperament is defined as a range of traits that characterize an individual over time and their “response to novelty in a non-social context” [4,9]. A species’ natural history contributes to temperament. In both species, calves and subadults are more active and playful, and mothers and calves have the closest social relationships [10,11]. Black rhino females form small, transient groups that share home ranges [11–13], whereas white rhino females and subadults form long-lasting companionships and larger transient groups of up to 14 individuals in the wild [10]. Adult male black rhinos tend to be solitary and only interact with conspecifics when mating or competing over a female or a territory [14]. Adult male white rhinos also are more solitary than females, with whom they typically only interact for breeding, but will interact transiently with other adult or subadult males living on the same territory [15]. Ex situ adult black rhinos are usually housed solitarily; female white rhinos are usually housed socially and males solitarily except for breeding purposes [16]. These differences in sociality between the species suggest black rhinos could show less affiliative temperaments than white rhinos.

Temperament can be assessed by looking at traits independently or in combination due to its complex nature [5]. Methods commonly used to assess temperament of wildlife include temperament surveys and novel object tests, among others [5]. Temperament surveys use the extensive knowledge of those familiar with the individuals to rate their traits [4,5,17]. While the subjective nature of surveys can challenge their validity, they offer the advantage of evaluating a large range of traits such as bold, active, aggressive, and sociable [4,18,19]. Novel object tests, which involve introducing an unfamiliar object into an animal’s environment and observing its reaction, best provide insights into exploration and fearful traits [4,9,20,21]. Temperament assessments comparing surveys and novel object tests frequently correlate, suggesting both help identify personality traits [4,22,23].

Despite the widespread use of surveys and novel object tests to study the temperament of wildlife in zoos [17, 23–28], their validity in assessing the temperament of black and white rhinos has not been thoroughly investigated. A study on black rhino temperament using surveys found a relationship between traits and breeding success in females [4]. However, temperament has never been investigated in white rhinos. The present study aims to identify demographic and environmental variables that relate to rhino temperament, the distinct insights provided by surveys and novel object tests and determine how they complement one another in assessing the temperaments of North American populations of black and white rhinos.

## Materials and methods

### Study population

All rhino-holding institutions in the United States and Canada were contacted to participate in this study. From these institutions, 56 agreed to participate in the temperament survey and 39 in the novel object test. This resulted in the participation of 42% of the black rhino and 58% of the white rhino ex situ populations in the United States and Canada at the time of the study. The study consisted of 50 black rhinos and 125 white rhinos (S1 Table) in human care, all > 2 yrs of age. Temperament surveys were collected for 49 (24M:25F) black rhinos and 32 (16M:16F) participated in novel object tests, resulting in 31 rhinos with both assessments. Surveys were completed for 125 (45M:80F) white rhinos, 66 (29M:37F) of which also participated in novel object tests. Most black rhinos (34/50; 68%) were housed individually, and most white rhinos (108/125; 86%) were housed socially with other rhinos. All rhinos were managed in accordance with the Association of Zoos and Aquariums standards for care. Black rhino diets generally included pelleted feed, hay, and browse, and white rhino diets consisted of pelleted feed, hay, and pasture grasses. This study was approved by IACUCs at George Mason University (#1833567), the Cincinnati Zoo and Botanical Garden Center for Conservation and Research of Endangered Wildlife (#22–173), and by individual participating institutions. Written consent was provided by each participating institution.

### Temperament surveys

Temperament surveys (Table 1) were sent to participating institutions to be completed via Qualtrics (Qualtrics.com) by keepers who worked with the rhino ≥6 months. Surveys were completed by 2 + keepers, except for 14 white rhinos with only 1 survey response. Keepers were asked not to discuss their responses with others. The survey assessed general behaviors, interactions with familiar people, interactions with strangers, and interactions with conspecifics (only for rhinos housed socially) on a 10-point sliding scale, from 0 (never) to 10 (always) (Table 1).

**Table 1:** Temperament surveys assessing the temperament of black (n = 49) and white (n = 125) rhinos in managed care based upon their general behaviors, interactions with conspecifics, familiar people, and strangers.

Behaviors	0 = never or 0% 10 = always or 100%	N.A.
**General Behaviors**	**0** **1** **2** **3** **4** **5** **6** **7** **8** **9** **10**	
**Active**: Moves frequently (patrols, walks a lot, active foraging)	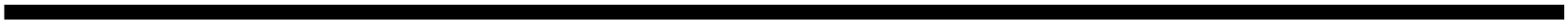	☒
**Calm**: Not easily disturbed by changes in the environment	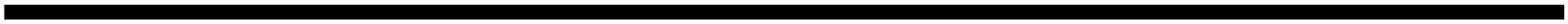	☒
**Investigative**: Readily approaches and explores changes in the environment	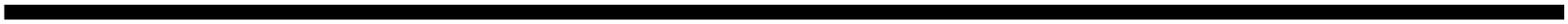	☒
**Sleep**: Time spent recumbent with head down during the day	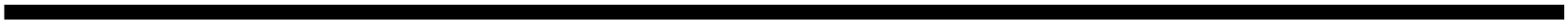	☒
**Stereotypies**: Exhibits stereotypic or unusual behaviors	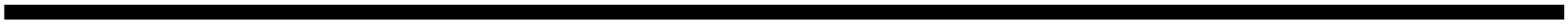	☒
**Interactions with Conspecifics**	**0** **1** **2** **3** **4** **5** **6** **7** **8** **9** **10**	
**Assertive**: Readily displaces conspecifics without contact aggression (vocalizations may occur)	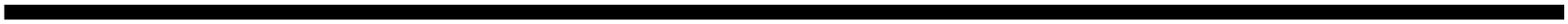	☒
**Chemosensory**: Urine spraying, dung kicking, scraping, investigating midden	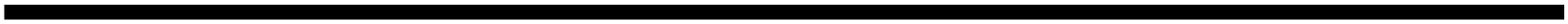	☒
**Conspecific affiliative**: Initiates and engages in prosocial, species appropriate behavior	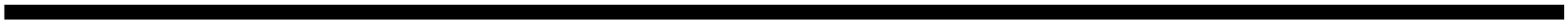	☒
**Conspecific aggressive**: Frequently reacts hostile, advances on, or displays aggressive vocalizations	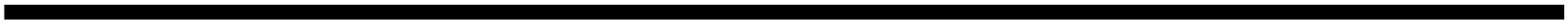	☒
**Conspecific fearful**: Retreats from and/or keeps distance from conspecifics	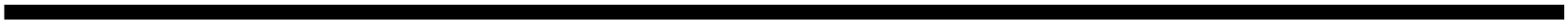	☒
**Food aggressive**: Becomes aggressive to (e.g., advances on or vocalizes) and/or displaces conspecific from food	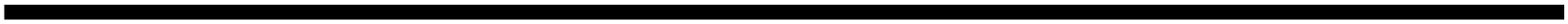	☒
**Novel object**: Dominates access to novel objects (e.g., toys or enrichment) with aggressive displays and/or body position	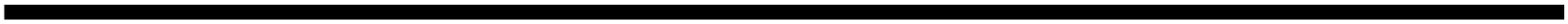	☒
**Preferred object**: Dominates access to preferred objects (e.g., toys or enrichment) with aggressive displays and/or body position	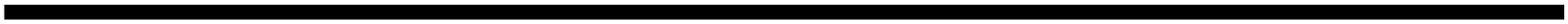	☒
**Social**: Initiates and seeks out close proximity to other rhinos	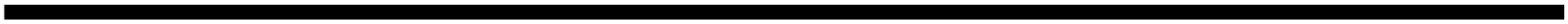	☒
**Vocal**: Frequently and readily vocalizes to conspecifics	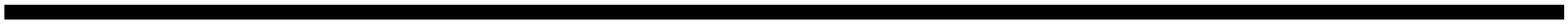	☒
**Interactions with Familiar people**	**0** **1** **2** **3** **4** **5** **6** **7** **8** **9** **10**	
**Familiar affiliative**: Initiates proximity: approaches readily and in a friendly manner (e.g., allows touching) to primary staff	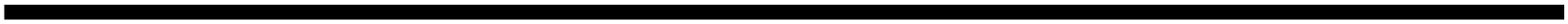	☒
**Familiar aggressive**: Frequently reacts hostile, strikes out or displays aggressive behavior or vocalizations to primary staff	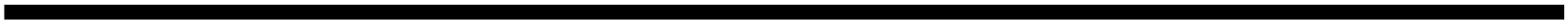	☒
**Familiar fearful**: Retreats from; keeps away from primary staff	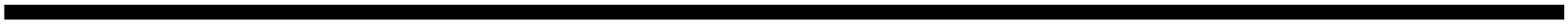	☒
**Responsive**: Responds cooperatively to requests from primary staff	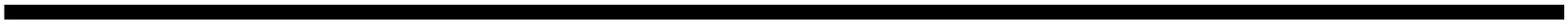	☒
**Interactions with Strangers**	**0** **1** **2** **3** **4** **5** **6** **7** **8** **9** **10**	
**Stranger affiliative**: Initiates proximity: approaches readily and in a friendly manner (e.g., allows touching) to strangers	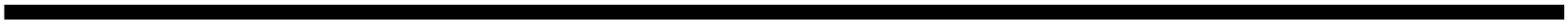	☒
**Stranger aggressive**: Frequently reacts hostile, strikes out or displays aggressive behavior or vocalizations to strangers	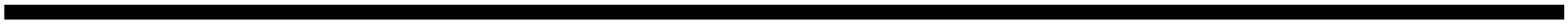	☒
**Stranger fearful**: Retreats from; keeps away from strangers	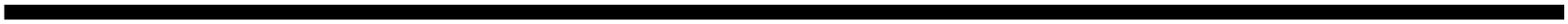	☒

The inter-rater reliability (IRR) for surveys was tested with the R package rcompanion [29] using Kendall’s *W* for every rhino with at least two raters. Kendall’s *W* was calculated for every behavior for each rhino. Results demonstrated high variability among raters for black and white rhinos. Thus, the median keeper rating was used in analyses to reflect the central tendency of each rhino’s rated behavior because it is robust to extreme ratings. Tables with Kendall’s *W* values are available for both species [see 30].

A Principal Component Analysis was conducted for each species using scaled median keeper ratings with the R package stats [31]. The PCA clustered median keeper ratings into principal components (PC) and included all behaviors, except those assessed only for socially-housed rhinos (Table 1). The number of PCs selected was based on the elbow method and a threshold of 65% explained variance [32]. Selected PCs and a wildlife literature review (Table 2) in which behaviors aligned with personality traits in previous studies provided the basis for dividing the behaviors rated in the survey into the following predominant personality traits: anthrophilic/-phobic, bold/fearful, sociable/aggressive with conspecifics, active/inactive, exploratory/avoidant. The PCs are subsequently referred to as ‘temperament profiles’.

**Table 2:** Literature review used to divide rated behaviors from the temperament survey into personality traits for black (n = 66) and white rhinos (n = 125).

Personality traits	Survey behaviors	Personality traits	Survey behaviors
**Anthrophilia**	Familiar affiliative [20] Stranger affiliative [20] Responsive [4,20]	**Anthrophobia**	Familiar aggressive [20] Familiar fearful [20] Stranger aggressive [20] Stranger fearful [20]
**Bold**	Calm [20] Investigative [20,21] Novel object [20,33]	**Fearful**	Conspecific fearful [20] Familiar fearful [20] Stranger fearful [20] Stereotypies [20] Vocal [34–36]
**Sociable**	Conspecific affiliative [20]	**Aggressive**	Conspecific aggressive [20]
**Active**	Active [20]	**Inactive**	Sleep [20]
**Exploratory**	Investigative [20] Novel object [20,33] Chemosensory [10,37]		

### Novel object test

Novel object tests were conducted with individual rhinos separated from conspecifics, except for females (n = 1 black rhino, n = 11 white rhinos) that were accompanied by their calves (S1 Table). The novel object, a cotton rope basket 34.8 cm x 24.9 cm x 20.0 cm (product #VT2101O3THB by Voten, China), was placed ~ 4.5m from the enclosure entrance. All baskets were washed with the same soap prior to the test to control for diverse olfactory responses. To maintain the novelty of the basket, the test was performed only once per rhino. The enclosure was chosen by the animal care staff. Due to the expected variability among institutions, ambient temperature, calf presence (yes/no), enclosure access (outdoor/indoor), and enclosure size (small, < 35.0 m^2^ / medium, 35.0–140.0 m^2^ / large, > 140.0 m^2^) were recorded before starting observations.

Observers used ZooMonitor (www.zoomonitor.org) to record the focal rhino’s response to the novel situation using an ethogram (Table 3), which included latency to approach the basket, state behaviors for which duration was recorded, event behaviors for which frequency was recorded, and distance to the object that was recorded at 1-minute intervals. Prior to collecting data, observers passed the IRR test at 95.2% for state behaviors and 88.5% for event behaviors using intraclass correlation coefficients (ICC). The observation session started as soon as the rhino was visible through the enclosure entrance, and its behavioral response (Table 3) was recorded continuously for 20 minutes.

**Table 3:** Novel object test assessed the temperament of black (n = 32) and white rhinos (n = 66) in North American zoos. Novel object ethogram was adapted from [4,10,11,38,39].

Behavior	Description
**Latency to approach**	Time (sec) from release into the enclosure to moving to within 2 meters of the novel object
**State behaviors (duration)**	
**Active**	Locomotion; including grazing and excluding pace; choose Stimulated or Unstimulated (see “Stimulated” below for definition)
**Gate wait**	Positioned next to a closed door or yard gate; within one body length; choose Stimulated or Unstimulated
**Inactive**	Not moving; includes standing, sleeping, resting, and voiding (other than next to the object); choose Stimulated or Unstimulated
**Stimulated (Active/Gate wait/Inactive)**	Head up (jaw line above leg fold in WR and above point of shoulder in BR), OR Tail curled (except during a void), OR Running (Active only); might include high step (Active and Gate wait only)
**Pace**	Repetitive walking, such that the same path is followed three or more times. Record it as pacing at the start of the third repetition
**Other**	Behavior not defined on ethogram
**Not visible**	Behavior of the individual not visible to observer
**Contact behaviors (event/all-occurrence)**	
**Attack**	Aggressively jabbing object with the horn
**Contact interaction**	Moving or touching object using any body part; exclude mouthing
**Non-contact behaviors (event/all-occurrence)**	
**Advance directed**	Move directly towards the object (within one body length) but does not make contact; excluding charge
**Charge**	Rapid advance towards the object (within one body length) but does not make contact
**Head fling**	Heads swing up and down rapidly toward object (within one body length) but does not make contact
**Other behaviors (event/all-occurrence)**	
**Olfactory – other**	Sniffing, includes flehmen (head up, opened mouth with curled upper lip)
**Olfactory – object**	Sniffing, includes mouthing (take object into mouth and then spit it out) and flehmen (head up, opened mouth with curled upper lip); on or within 2 meters from the object
**Retreat**	Backing away or running away from the object, either after contact or when within one body length
**Pant**	Short, wheezy, and quick breaths from chest
**Snarl**	Growl or rumbling
**Snort**	Nasal inhalation or exhalation
**Whine**	Thin cry; pitch rises and falls
**Distance (1-minute interval sampling)**	
**Distant**	More than 2 meters from the object
**Near**	Within 2 meters from the object

Previous studies on animal temperament and personality (Table 4), and their categorization of behaviors were used to guide how the behaviors from the novel object test from the present study were divided into personality traits including bold/fearful, active/inactive, exploratory/avoidant, and sociable/aggressive with conspecifics (Table 4).

**Table 4:** Literature review used to divide behaviors from novel object test into personality traits for black (n = 32) and white rhinos (n = 66).

Personality traits	Novel object test behaviors	Personality traits	Novel object test behaviors
**Bold**	Short latency to approach [33,37] Advance directed [33,37] Unstimulated [33,37] Near [33,37]	**Fearful**	Long latency to approach [33,37] Stimulated [33,37] Gate wait [4,26,33] Pace [26,33] Attack [21,33] Whine [10,38] Pant [10,34,38]
**Active**	Active [20]	**Inactive**	Inactive [20]
**Exploratory**	Contact interaction [20,37] Olfactory – object [20,37] Olfactory – other [20,37]	**Avoidant**	Retreat [33] Snarl [10,38] Snort [10,38]
**Sociable**	Pant [10,34,38]		

Frequencies for all-occurrence and interval behaviors were calculated using the number of occurrences divided by the total session duration in minutes excluding any time not visible. Proportions for state behaviors were calculated using the duration in seconds divided by the total session duration in seconds. Behaviors that occurred in <1% of rhinos during the novel object test were not modeled to maintain robust sample sizes. These behaviors included snort, snarl, whine, charge, and head fling in both species, pant calls and attack in black rhinos, and retreat and pace in white rhinos. Behavioral richness was calculated by adding up all the behaviors displayed by each rhino. The Shannon diversity index was calculated using the vegan R package using frequencies for all-occurrence and proportions for state behaviors [40,41].

### Data analysis

Analyses were conducted using R version 4.3.2 [31]. Mann-Whitney U tests were conducted on non-normally distributed data to compare results between species using median keeper ratings from the survey, and behavioral richness and diversity index from the novel object test. The data for the novel object test behaviors showed overdispersion. Therefore, negative binomial regressions, which account for overdispersion, were used to model novel object test behaviors, duration of state and count for interval and all-occurrence, to compare results between species. To account for variability in novel object test session length due to time not visible, the log-transformed total duration (in minutes for all occurrence, in seconds for state behaviors) was used as an offset term in the model. DHARMa package was used to run diagnostics to ensure overdispersion is addressed in the model and that residuals follow a normal distribution [31,42]. All other analyses were conducted within each species. The R scripts containing the code used for data analysis are available online in a GitHub repository (https://github.com/AIRS-Rhino/Rhino-Temperament/). A threshold of *p* < 0.05 was used to determine statistical significance.

### Temperament survey models

PCA temperament profile scores, which are continuous variables with a normal distribution, were modeled with sociality (individual/socially housed), birth type (wild/captive), age, and sex as fixed effects and institution as a random effect using linear mixed models [31,43]. The random effect of institution was only included for the temperament profile models when it significantly contributed to the model. Significant contribution of institution was determined by performing an ANOVA to compare the models with and without the random effect (*p* < 0.05). Starting with the full model, the `drop1()` function from the stats R package was used to find the best model [31]. The DHARMa package was used to run diagnostics to ensure residuals were normally distributed and adequate model fit [31,42]. The most parsimonious model within 2 units of the lowest AIC was selected [33]. Detailed tables with full models and all models within 2 AIC units of the lowest model are available [see 31].

### Novel object test models

The behaviors from the novel object test, duration of state behaviors and count for interval and all-occurrence behaviors, were modeled with age, sex, sociality, enclosure access, enclosure size, temperature, calf presence (WR only), and birth type (wild/captive, WR only) as fixed effects and institution as a random effect using negative binomial mixed regression models to address the overdispersion [44]. To account for variability in session length due to time not visible, the log-transformed total duration (in minutes for all occurrence, in seconds for state behaviors) was used as the offset term in the model. The random effect of institution was only included for the novel object test models when it had a significant contribution. Significance was determined by performing an ANOVA to compare the models with and without the random effect (*p* < 0.05). Starting with the full model, the `drop1()` function from the stats R package was used to find the best model [31]. DHARMa package was used to run diagnostics to ensure overdispersion is addressed, that residuals follow a normal distribution, and adequate model fit [31,42]. The most parsimonious model within 2 units of the lowest AIC was selected [33]. Detailed tables with full models and all models within 2 AIC units of the lowest model are available [see 31].

### Correlation between surveys and novel object tests

To reduce multicollinearity within each temperament assessment, correlations were conducted to consolidate behaviors prior to the between-assessment correlation analysis. Pearson’s correlations were conducted between behaviors using median keeper ratings from the temperament survey and Spearman’s correlations were used for the novel object test behaviors using frequencies and proportions.

Multiple highly correlated (r ≥ |0.60|) behaviors were removed or modified prior to comparisons between temperament assessments (S2-S4 Tables). Spearman’s correlations were performed to determine the relationship between the temperament survey and novel object tests using the retained/consolidated behaviors. PCA temperament profiles and median keeper ratings were used for the survey, and the frequencies and proportions of recorded behaviors were used for the novel object test. The *p*-values for the Spearman’s correlations between assessments were adjusted using the Benjamini-Hochberg method to control the false discovery rate (fdr) for multiple comparisons [45].

## Results

### Temperament survey

Black rhinos were rated more stereotypic and aggressive towards familiar people and strangers than white rhinos (*p* < 0.05). Conversely, white rhinos were rated calmer, more vocal, and more affiliative towards strangers (*p* < 0.05). No other median keeper ratings differed between the two species (*p* > 0.05).

**Fig 1:**
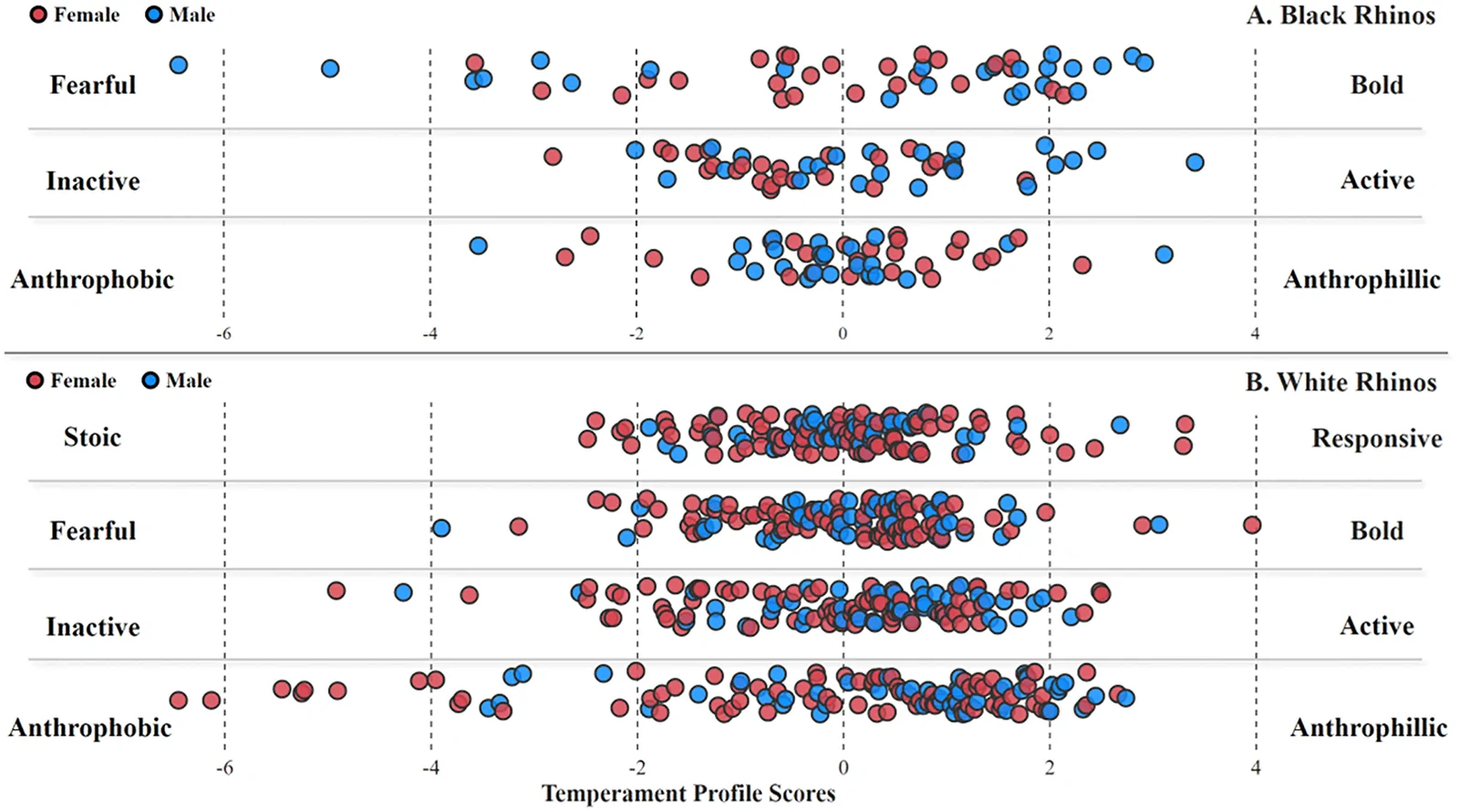
Temperament profile scores. Each point represents a temperament profile score for individual (A) black and (B) white rhino from PCA of median keeper ratings of 12 rated behaviors on temperament surveys. Females are in red and males in blue.

The PCA yielded three PCs (Table 5; Fig 1A; 65.1% explained variance) for black rhinos and four PCs (Table 5; Fig 1B; 69.2% explained variance) for white rhinos. PCs corresponded to the following temperament profiles: bold/fearful, active/inactive, and anthrophilic/-phobic in both species, and additionally, reactive/stoic in white rhinos (Table 5). The rated behaviors with the highest loadings contributing to each PC (temperament profile) were used to describe them (Table 5).

**Table 5:** Principal Component Analysis loadings. Loadings of each behavior from temperament surveys with principal components (PC) explaining ≥65% variance. Values in bold depict the largest loadings in magnitude within each PC.

	Black rhino			White rhino			
	**PC1**	**PC2**	**PC3**	**PC1**	**PC2**	**PC3**	**PC4**
	**Bold/Fearful**	**Active/Inactive**	**Anthrophilic/-Phobic**	**Anthrophilic/-Phobic**	**Active/Inactive**	**Bold/Fearful**	**Reactive/Stoic**
Active	0.08	**0.65**	0.02	−0.05	**0.63**	−0.05	−0.07
Calm	**0.38**	−0.01	0.02	0.17	−0.20	**0.46**	−0.12
Investigative	0.28	0.14	**−0.43**	0.18	**0.40**	**0.44**	0.14
Sleep	0.00	**−0.51**	0.00	0.06	**−0.57**	0.05	0.34
Stereotypies	−0.01	**0.39**	0.00	−0.21	−0.02	0.26	**0.57**
Familiar affiliative	0.32	−0.25	−0.31	**0.41**	0.10	−0.16	0.31
Familiar aggressive	−0.28	0.21	**−0.40**	**−0.37**	0.01	**0.44**	−0.06
Familiar fearful	**−0.37**	−0.05	0.17	−0.34	−0.02	−0.17	0.30
Responsive	0.29	−0.11	−0.39	0.22	0.16	0.06	**0.49**
Stranger affiliative	**0.41**	0.04	0.21	**0.42**	0.08	0.15	0.10
Stranger aggressive	−0.31	0.00	**−0.57**	**−0.37**	0.07	0.35	0.03
Stranger fearful	**−0.33**	0.16	−0.06	−0.33	0.18	−0.36	0.29
*Variance (%)*	*38.9*	*14.6*	*11.6*	*33.6*	*15.2*	*10.8*	*9.6*

In black rhinos, PC1 (range: −6.43 to 2.93; Fig 1A) described bold/fearful tendencies with positive loadings indicative of bold (calm, stranger affiliative) and negative loadings indicative of fearful (familiar and stranger fearful) rhinos (Table 5). PC2 (range: −2.80 to 3.42; Fig 1A) represented active/inactive traits, with positive loadings indicative of active and stereotypic rhinos, and negative loadings represented inactive (sleep) rhinos (Table 5). PC3 (range: −2.80 to 3.42; Fig 1A) indicated anthrophilic/-phobic tendencies, with positive loadings on PC3 reflecting anthrophilic and negative loadings anthrophobic rhinos that were rated as aggressive toward familiar people and strangers, yet investigative (Table 5).

In white rhinos, PC1 (range: −3.89 to 3.97; Fig 1B) explained anthrophilic/-phobic tendencies, with positive loadings representing anthrophilic rhinos (familiar and stranger affiliative), while negative loadings were for anthrophobic (familiar and stranger aggressive) rhinos (Table 5). PC2 (range: −4.91 to 2.51; Fig 1B) was characterized as active/inactive. Positive loadings represented active and investigative rhinos and negative loadings represented inactive (Table 5) individuals. PC3 (range: −3.89 to 3.97; Fig 1B) indicated bold (calm, investigative, familiar aggressive) individuals and white rhinos with negative loadings fell on fearful end of the spectrum (Table 5). Finally, PC4 (range: −2.48 to 3.32; Fig 1B) described reactive/stoic tendencies with positive loadings indicative of rhinos that were stereotypic and responsive to familiar people (Table 5).

**Fig 2:**
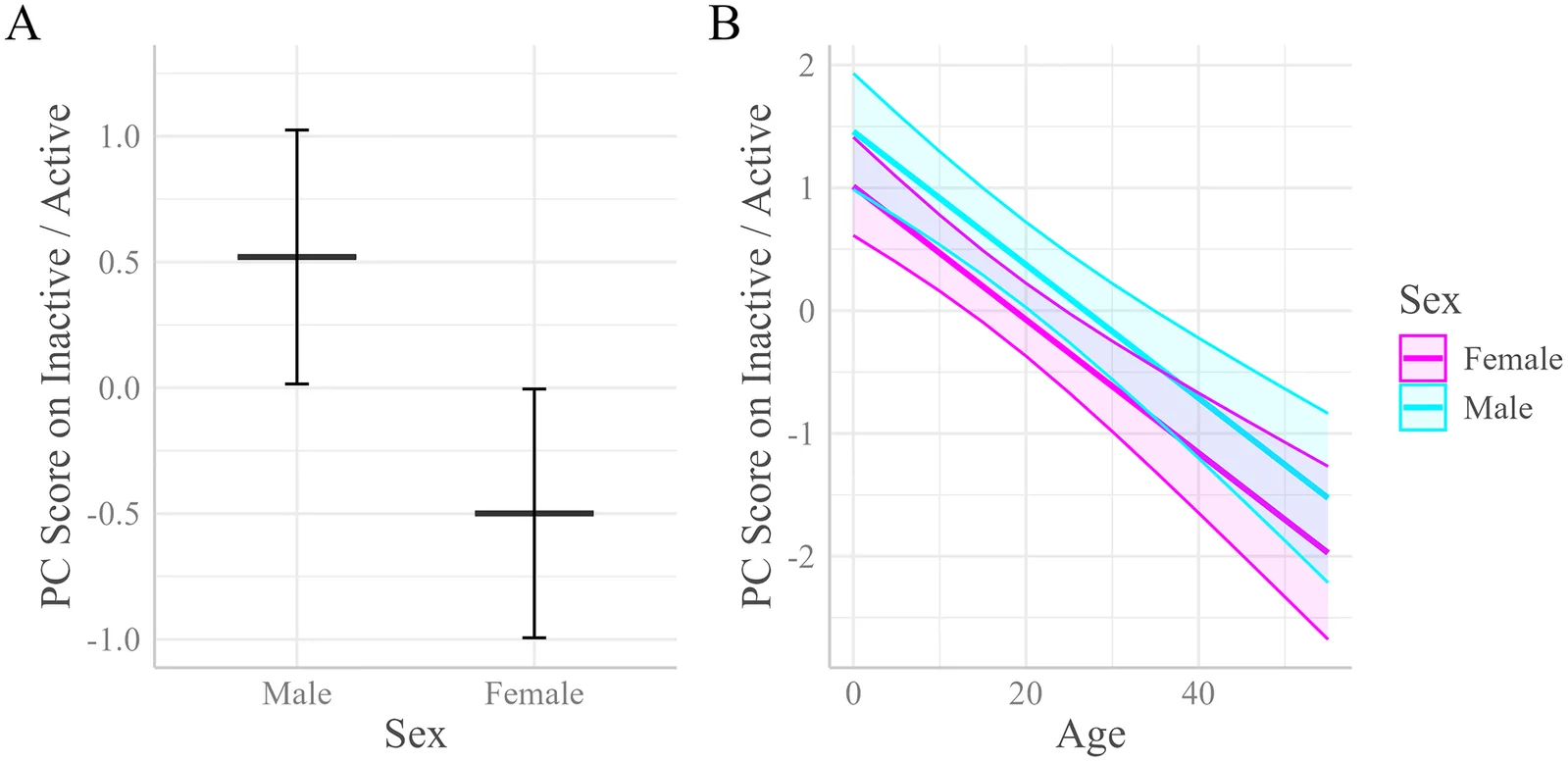
Best models for the active/inactive temperament profile (PC2) using linear mixed models. (A) Scores for black rhinos. (B) Scores by age and sex for white rhinos.

Some temperament profiles were influenced by sex and age in rhinos. Female black and white rhinos were rated as less active than males (Fig 2A-2B; Table 6). In white rhinos, older individuals were rated as less active (Fig 2B; Table 6). Ratings of anthrophilic/phobic and reactive/stoic were influenced by the institution at which white rhinos were housed (Table 6).

**Table 6:** Best models for black (n = 49) and white (n = 125) rhinos investigating demographic variables that impact temperament profiles (PCs).

Response variable	Model structure	df	AIC	∆AIC	*p*-value
Black rhino					
PC1, Bold/fearful	~ 1	2	217.44	0.00	**–**
PC2, Active/inactive	~ sex	3	163.23	0.00	**sex: 0.006**
PC3, Anthrophilic/-phobic	~ 1	2	158.21	0.00	**–**
White rhino					
PC1, Anthrophilic/-phobic	~ 1 + (1|institution)	5	516.72	0.00	–
PC2, Active/inactive	~ sex + age	4	391.13	0.00	**age < 0.001, sex: 0.021**
PC3, Bold/fearful	~ 1	2	390.53	0.00	**–**
PC4, Reactive/stoic	~ 1 + (1|institution)	3	338.33	0.00	**–**

df, degrees of freedom; AIC, Akaike Information Criterion; ∆AIC, difference from the lowest AIC model. Values in bold indicate significance of *p* < 0.05.

### Novel object test

All black rhinos and all but one white rhino approached the basket. Black rhinos paced snorted, charged, retreated, advanced directly toward, physically contacted, engaged in olfactory behaviors and were near the basket more frequently than white rhinos (*p* < 0.05), while white rhinos vocalized pant calls more frequently than black rhinos (*p* < 0.05). Black rhinos had a higher behavioral diversity index than white rhinos (*p* < 0.05).

**Fig 3:**
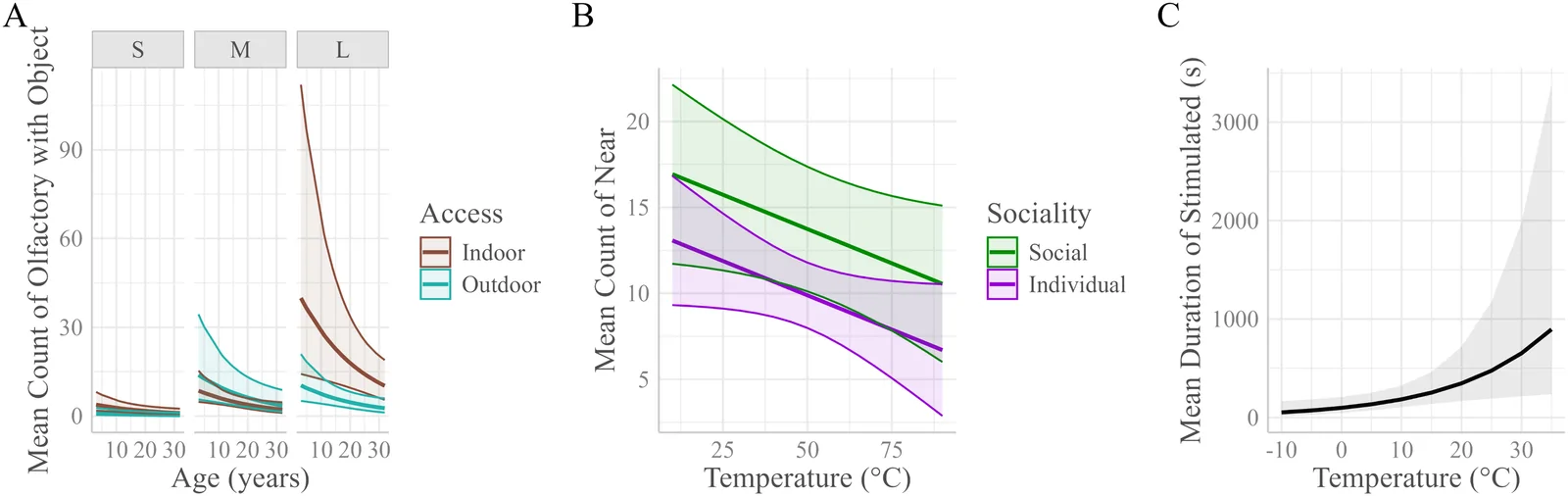
Best models for novel object test behaviors in black rhinos using negative binomial regression models. (A) Olfactory behavior toward the novel object by age, enclosure size and indoor/outdoor access. (B) Intervals near the novel object by temperature, separated by sociality. (C) Duration of time stimulated by temperature.

During the novel object test, black rhinos displayed between 5 and 14 behaviors (*M* = 8.63) and their behavioral diversity index (BDI) ranged from 1.26 to 2.35 (*M* = 1.77). Black rhinos with a higher BDI score tended to show their behaviors more evenly. Older black rhinos displayed olfactory behaviors toward the basket less frequently (Table 7; Fig 3A). Additionally, olfactory behaviors toward the basket occurred most frequently in large, indoor enclosures (Fig 3A). Black rhinos spent less time near the basket (Fig 3B) and more time stimulated as temperature increased (Table 7; Fig 3C).

**Table 7:** Best models for black (n = 32) and white (n = 66) rhinos for novel object test.

Response variable	Model structure	df	AICc	∆AIC	Overall *p*-value
Black rhino					
Retreat	~ 1	2	98.50	0.60	–
Olfactory w/object	~ age + access * size	7	177.18	0.00	age: 0.009, access*size: 0.004
Olfactory w/other	~ size	4	122.48	1.23	size: 0.076
Advance directed	~ 1	2	144.38	0.00	–
Contact interaction	~ 1	2	196.32	0.00	–
Near	~ sociality + temperature	4	195.82	0.00	sociality: 0.054, **temp: 0.047**
Stimulated	~ temperature	3	383.54	0.00	**temp: 0.040**
Active	~ sex	3	438.53	0.00	sex: 0.190
Inactive	~ 1	2	475.05	0.00	–
White rhino					
Pant	~ sex + calf presence + sex * calf presence	4	171.97	1.19	**sex: 0.003, calf: 0.006**
Olfactory w/object	~ age + sex + sociality + temperature	6	267.54	0.00	**age: 0.009, sex: 0.005, sociality: 0.011, temp<0.001**
Olfactory w/other	~ size	4	278.27	0.00	**size: 0.030**
Advance directed	~ access	3	223.02	0.46	access: 0.054
Contact interaction	~ age + temperature	4	305.23	0.00	**age: 0.017, temp<0.001**
Attack	~ access	3	84.98	0.46	**access: 0.024**
Near	~ size	4	385.43	0.44	**size: 0.002**
Stimulated	~ 1	2	684.21	1.16	–
Active	~ sociality + age	4	868.30	0.37	**sociality: 0.020, age: 0.005**
Gate wait	~ 1	2	292.83	0.00	**–**

df, degrees of freedom; AIC, Akaike Information Criterion; ∆AIC, difference from the lowest AIC model. Values in bold indicate significance of *p* < 0.05.

**Fig 4:**
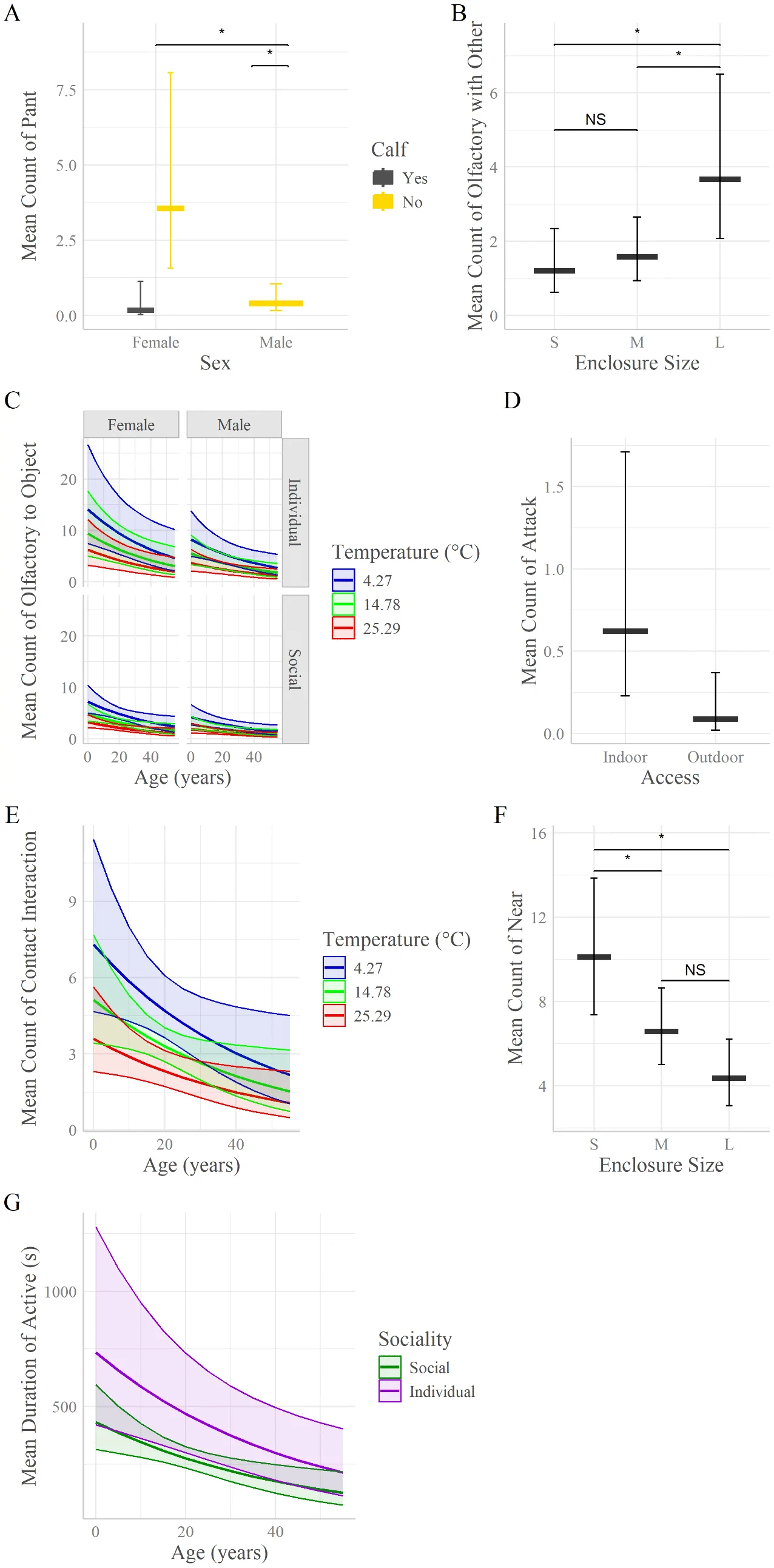
Best models for novel object test behaviors in white rhinos using negative binomial regression models. (A) Mean count of pant calls by sex and calf presence in females. (B) Mean count of olfactory behavior to the basket as rhinos age by sex (left and right panels), sociality (top and bottom panels), and temperature in Celsius. (C) Mean count of olfactory behavior to other than the basket as enclosure size increases. (D) Mean count of contact interactions with the basket as rhinos age by temperature. (E) Mean count of attacks on the basket in indoor v. outdoor enclosures. (F) Mean intervals near the novel object by enclosure size. (G) Duration spent active as rhinos age by sociality.

White rhinos displayed between 4–12 behaviors (*M* = 7.86) and the behavioral diversity index ranged from 0.82 to 2.24 (*M* = 1.60). Females vocalized pant calls twice as often compared to males and three times less frequently when a calf was present (Table 7; Fig 4A). Olfactory behaviors towards the basket were less frequent in older white rhinos and as temperature increased (Fig 4B). Females displayed olfactory behaviors with the basket twice as much compared to males, and individually-housed white rhinos twice as often as those socially-housed (Fig 4B). Olfactory behavior towards something other than the basket occurred more frequently in large enclosures than small and medium enclosures (Fig 4C). Older rhinos and higher temperatures were associated with lower frequencies of contact interactions with the basket (Fig 4D). White rhinos tested in indoor enclosures were seven times more likely to attack the basket than in outdoor enclosures (Fig 4E). Rhinos in large enclosures were less likely to be near the basket (Fig 4F). Lastly, white rhinos housed individually were twice as active as rhinos housed socially, and older rhinos spent less time active (Fig 4G).

### Correlations between temperament survey and novel object test

Many of the behaviors measured during the novel object test were significantly related to median keeper ratings and PCA temperament profiles from the survey, particularly in black rhinos (*p* < 0.05; Table 8). For instance, median rating for “vocal” was negatively related to proportion of time near the basket and latency to approach (*p* < 0.05). Median rating for “investigative” was positively correlated with frequency of olfactory behaviors toward the novel object, contact interactions, and proportion of time near the novel object (*p* < 0.05). Median rating for “fearful toward conspecifics” was positively correlated with proportion of time pacing and negatively related to latency to approach (*p* < 0.05). Median rating for “calm” was positively correlated with latency to approach (*p* < 0.05). Lastly, median rating for “active” was positively correlated with olfactory behaviors toward something other than the novel object (*p* < 0.05).

**Table 8:** Black rhino Spearman’s correlation between temperament profiles and median keeper ratings from the temperament surveys (rows) and novel object test behaviors (columns).

			Novel object test behaviors										
Survey behaviors	Personality traits (+/- spectra)		Active	Bold			Bold & Exploratory				Exploratory		
			Active	Gate wait	Stimulated	Pace	Advance direct	Latency approach	Near	Retreat	Contact interaction	Olfactory object	Olfactory other
**Active**	**PC2: active/inactive**	**0.37**	-0.04	0.15	0.07	0.28	-0.17	-0.13	-0.18	-0.10	0.28	0.31
	**Active**	0.34	0.06	0.15	0.01	0.26	-0.29	-0.02	-0.12	0.08	0.28	**0.42**
**Anthrophilic**	**PC3: anthrophilic/phobic**	-0.11	0.26	-0.02	0.04	-0.13	-0.03	-0.13	-0.23	-0.28	-0.24	-0.35
	**Human affiliative**	-0.32	0.20	-0.17	-0.17	0.06	0.09	0.20	-0.17	0.01	-0.10	0.27
	**Human aggressive**	0.33	-0.31	0.27	0.05	0.10	-0.02	-0.16	0.06	0.05	0.13	0.14
	**Responsive**	-0.25	-0.06	-0.18	-0.02	-0.05	0.00	0.22	-0.13	0..08	0.01	0.15
**Anthrophilic & Bold**	**Human fearful**	0.21	**-0.39**	0.04	0.13	-0.04	-0.15	-0.09	0.11	0.14	0.18	-0.11
**Bold**	**PC1: bold/fearful**	-0.35	0.25	-0.23	-0.28	-0.01	0.22	0.28	-0.23	0.10	0.02	0.17
	**Calm**	-0.30	0.18	-0.18	-0.34	-0.04	**0.42**	0.15	-0.25	0.06	0.00	0.04
	**Conspecific fearful^†^**	0.72	0.04	-0.12	**0.82**	-0.11	**-0.89**	-0.70	-0.07	-0.02	0.02	-0.16
	**Stereotypies**	0.05	**-0.38**	-0.01	0.07	-0.17	0.08	-0.18	-0.32	**-0.38**	0.00	-0.17
	**Vocal^†^**	**0.76**	0.49	0.41	**0.91**	0.33	**-0.91**	**-0.87**	0.53	0.07	-0.56	-0.62
**Bold & Exploratory**	**Investigative**	-0.20	0.09	-0.09	-0.30	0.15	0.23	**0.42**	-0.21	**0.45**	**0.41**	0.13
	**Novel object^†^**	0.11	0.47	0.71	0.04	**0.96**	0.00	-0.19	0.65	0.70	-0.59	-0.49
**Exploratory**	**Chemosensory^†^**	-0.11	-0.55	**-0.81**	-0.15	-0.39	-0.04	0.29	**-0.89**	0.14	**0.86**	0.67
**Sociable**	**Conspecific affiliative^†^**	0.00	-0.45	-0.59	0.26	-0.25	-0.50	-0.07	-0.33	-0.14	0.25	0.14
	**Conspecific aggressive^†^**	0.36	0.59	**0.77**	0.48	0.54	-0.29	-0.54	0.63	0.29	-0.71	-0.38

Values in bold indicate significance of raw p < 0.05. After fdr correction over 187 tests, no p-values remained significant. † Small sample size (n = 7).

Conversely in white rhino (Table 9), the anthrophilic/phobic (PC1) temperament profile was negatively related to the frequency of pant calls during the novel object test (*p* < 0.05). The bold/fearful (PC3) temperament profile was negatively related to the proportion of time near the basket (*p* < 0.05). Further analysis on this correlation using the interaction of near the basket and enclosure size in a linear regression model demonstrated that fearful white rhinos were not more likely to be near the object when enclosure size was considered (*p* > 0.05). Median ratings for “fearful toward strangers” and “stereotypies” were positively correlated with the proportion of time waiting at the gate during the novel object test. Median rating for “affiliative towards familiar people” was negatively related to the frequency of pant calls (*p* < 0.05).

**Table 9:** White rhino Spearman’s correlation between temperament profiles and median keeper ratings from the temperament surveys (rows) and novel object test behaviors (columns).

			Novel object test behaviors									
**Survey behaviors**	**Personality traits** **(+/-) spectra**		**Active**	**Bold**			**Bold & Exploratory**			**Exploratory**		**Sociable & Bold**
			**Active**	**Gate wait**	**Stimulated**	**Attack**	**Advance direct**	**Latency approach**	**Near**	**Contact interaction**	**Olfactory other**	**Pant**
	**Active**	**PC2: active/inactive**	0.13	−0.20	0.19	−0.17	0.09	0.05	−0.09	0.01	−0.06	−0.04
		**Active**	0.11	−0.23	0.10	−0.18	−0.07	−0.23	−0.05	−0.08	0.04	0.04
	**Anthrophilic**	**PC1: anthrophilic/-phobic**	0.02	−0.20	−0.21	−0.16	0.02	0.22	−0.09	0.04	−0.02	**−0.26**
		**Human aggressive**	−0.02	0.13	0.11	0.13	−0.21	−0.22	−0.10	−0.13	0.05	**0.25**
		**Familiar affiliative**	0.03	−0.07	0.01	−0.14	0.07	0.17	0.01	0.21	−0.09	**−0.32**
		**Responsive**	−0.06	−0.12	−0.17	−0.19	−0.01	−0.05	−0.16	0.12	−0.12	−0.19
		**Stranger affiliative**	0.00	−0.14	−0.20	−0.18	0.01	0.14	−0.20	−0.04	−0.07	−0.12
	**Anthrophilic & Bold**	**Familiar fearful**	−0.17	0.19	0.14	0.03	0.07	−0.01	0.15	−0.04	−0.08	0.17
		**Stranger fearful**	0.03	**0.27**	**0.29**	0.08	0.17	−0.15	0.05	0.16	−0.04	0.15
	**Bold**	**PC3: bold/fearful**	0.05	−0.05	−0.05	0.16	−0.20	−0.09	**−0.29**	−0.06	−0.06	0.07
		**Conspecific fearful**	0.17	**0.43**	0.19	0.03	0.08	−0.01	−0.10	−0.05	0.10	0.21
		**Stereotypies**	0.05	**0.27**	0.05	0.10	−0.03	−0.04	−0.10	0.00	0.01	0.11
	**Bold & Exploratory**	**Investigative**	0.15	−0.08	0.13	0.08	0.16	0.21	−0.21	0.14	−0.06	0.01
		**Novel object**	−0.03	0.14	0.15	0.17	−0.01	−0.07	0.05	**0.27**	−0.20	0.18
	**Exploratory**	**Chemosensory**	0.07	0.03	0.10	−0.13	−0.05	0.07	−0.11	−0.03	0.00	−0.10
	**Reactive**	**PC4: reactive/stoic**	−0.20	0.19	0.00	0.00	0.09	0.00	−0.08	0.20	−0.12	−0.03
	**Sociable**	**Conspecific affiliative**	0.03	−0.03	0.19	0.13	0.21	0.00	0.08	0.14	0.05	0.12
		**Conspecific aggressive**	−0.02	−0.05	0.01	0.21	−0.03	−0.17	0.00	0.25	−0.03	−0.05
	**Sociable & Bold**	**Vocal**	0.04	0.21	0.14	−0.02	0.08	0.04	−0.02	−0.12	0.03	**0.34**

Values in bold indicate significance of *p* < 0.05. After fdr correction over 187 tests, no *p*-values remained significant.

## Discussion

This study is the first to compare different assessments of temperament in both African rhino species. The multidimensionality of the principal components analysis and the variation in behaviors during the novel object test that were indicative of boldness and exploration demonstrated the complexity of rhino temperaments in both species. Although keeper ratings varied between species, the PCAs were able to consolidate similar temperament profiles for both species of bold/fearful, active/inactive, and anthrophilic/-phobic. Temperament of African rhinos was related to age, sex and/or institution in both assessment tools. Thus, these factors need to be considered to properly interpret rhino temperament assessments. With respect to the novel object test, enclosure parameters contributed to the rhino’s response to the basket, which could aid our understanding of the behavioral plasticity of African rhinos. Identifying rhino temperament via surveys and/or novel object tests can facilitate personalized care of each rhino to enhance positive wellbeing of these individuals.

### Rhino temperament complexity

There were differences in temperament by species. In the temperament surveys, black rhinos were rated by keepers as more stereotypic and aggressive than white rhinos, whereas white rhinos were rated as bolder, calmer, and more affiliative. Aligning with the survey results, novel object test behaviors demonstrated that black rhinos were more fearful (e.g., more time spent pacing and higher frequencies of retreat) than white rhinos. This demonstrates both assessment tools can distinguish bold/fearful tendencies.

For the anthrophilic/-phobic temperament profile, 62% of white rhinos and 53% of black rhinos fell on the anthrophilic side of the spectrum, suggesting white rhinos were more anthrophilic. Additionally, black rhinos were rated as more aggressive towards strange or familiar people, whereas white rhinos were rated as more affiliative towards strangers. These positive reactions to humans by white rhinos support descriptions of these rhinos as more anthrophilic [20]. These findings were consistent with rhino behavior in the wild that reveal black rhinos display threats to humans and are often considered anxious animals because of their intensified flight response in the presence of humans compared to other rhino species [11,14].

Although rhinos could be described in popular culture as characterless, lazy, or even aggressive, results of this study revealed complex temperaments among individuals. A study by Carlstead et al. (1999) demonstrated the complexity of black rhino temperament by clustering 14 traits into groups related to dominance, fear, olfactory behaviors, and chasing/stereotypy/mouthing [4]. In the present study, the survey rated behaviors clustered into temperament profiles related to bold/fearful, active/inactive, and anthrophilic/-phobic traits, plus an additional temperament profile related to reactive/stoic traits in white rhinos. Only the dimensions of fearfulness in Carlstead et al. (1999) and bold/fearful in the present study were similar between the two studies, in which different ethograms were used [4]. For example, the present study included traits related to interactions with familiar people and strangers (Table 1), whereas Carlstead et al. (1999) monitored “allows touching” and “approaches to call” to assess rhino responsiveness to the rater only [4]. Nonetheless, temperament assessments in other species, such as horses (*Equus caballus*), domestic cattle (*Bos taurus)*, and polar bears (*Ursus maritimus*), often measure exploration, boldness, and activity despite differences in ethograms [46–48]. For instance, a study on snow leopards (*Uncia uncia*) grouped behaviors similarly to the present study: active/vigilant, calm/self-assured, timid/anxious, and friendly to humans [23]. Complexity within these common temperament profiles is demonstrated by the broad range of temperament scores in the present study, especially for bold/fearful (PC1, −6.43 to 2.93) in black rhinos and anthrophilic/-phobic (PC1, −6.44 to 2.72) in white rhinos. These data reinforce that not all rhinos are alike or will likely respond similarly to their social and physical environment. Novel object tests also can reveal temperament complexity [4,47]. For instance, no rhino had the same behavioral diversity score, meaning each rhino displayed a unique set of behaviors at different rates in response to the novel basket. African rhinos also demonstrated the flexibility of their temperament expression as behavior was associated with different environmental conditions.

### Correlation between temperament survey and novel object test

Temperament surveys enable the assessment of a variety of traits using the expert knowledge of raters [5,21]; meanwhile, novel object tests are commonly used to determine exploration/avoidance [5,19] and boldness/fearfulness traits assessed in novel situations in animals [19,21,33]. In the present study, comparison between these two assessment tools demonstrated a potential overlapping utility in understanding temperament traits, particularly for black rhinos, when considering the raw *p*-values. It is worth noting that these are exploratory analyses and more experimental tests are needed to verify how these preliminary findings relate to our understanding of rhino temperament and wellbeing.

Many survey results and novel object measurements of activity and exploration were correlated in black rhinos. For example, positive correlations were detected between investigative survey ratings and contact interactions with the basket, chemosensory ratings and olfactory behaviors with the basket, active ratings and olfactory behaviors to other than the novel object, and the active/inactive (PC2) temperament profile with time active during the novel object test. These results align with another study that shows activity can be an indicator of exploration [20]. Additionally, all behaviors measured from the novel object test were correlated with at least one survey behavior or PCA temperament profile, suggesting novel object tests could be an effective assessment for unfamiliar people with individual black rhinos to gain insight into their temperament.

In contrast, fewer behaviors associated with the novel basket were related to survey rated behaviors or PCA temperament profiles of white rhinos. Nonetheless, positive correlations were detected between novel object ratings from the survey and contact interactions with the basket, stereotypic ratings and time gate waiting, and vocal ratings and pant calls during the novel object test, demonstrating that these traits could be assessed with a novel object test. Additionally, pant calls, which rhinos use to seek contact with conspecifics or to communicate about a disturbance [10,34,38], stood out for multiple associations with behaviors related to anthrophilia. White rhinos rated as anthrophobic (PC1), less affiliative to familiar people and more aggressive to familiar people and strangers, had a higher frequency of pant calls during the novel object test. This suggests that panting might have indicated distress by rhinos over separation from conspecifics during the novel object test. White rhinos that gate waited more during the novel object test were rated as more fearful of conspecifics and strangers, and as displaying more stereotypies, thus further supporting that white rhinos may have been uncomfortable during the perceived risky situation of being separated from conspecifics. Stranger fearful white rhinos also were more stimulated during the novel object test. Overall, these findings imply white rhinos might respond more to the separation from conspecifics than the novel basket.

Novel object tests are widely used to monitor behavioral traits related to exploration/avoidance and boldness/fearfulness [19]. The present study demonstrates that these tests may be useful not only for evaluating these established personality traits in both rhino species, but also for assessing less commonly examined traits, such as sociability in both species and anthrophilia in white rhinos. For instance, the positive association between conspecific aggressive ratings from the survey and time black rhinos were stimulated during the novel object test suggested sociable/aggressive tendencies towards conspecifics may be assessed by zoos with a novel object test. Similarly, pant calls by white rhinos during the novel object test were positively related to aggressiveness towards humans and negatively associated with the anthrophilic/-phobic temperament profile. Because none of these initial findings were significant after fdr correction, additional experiments are needed to test these relationships and determine if novel object tests can be used to predict these personality traits.

### Effect of demographic and environmental factors on temperament

Sex was associated with temperament profiles generated from the survey in both species, whereas institution was associated with only white rhino temperament profiles. Responses to the novel basket were associated with age, ambient temperature and enclosure size where the test was conducted in both species. Additionally, sex, calf presence and sociality were related to white rhino behaviors.

Males of both species were rated as more active by keepers. In the wild, both black and white rhinos are territorial [10,11,14,23,49], with males frequently patrolling their territory [10,11]. Even though ex situ males do not need to compete for territories, the innate desire to patrol and mark their territory likely contributes to higher activity levels than non-territorial females [10,11]. Younger white rhinos were rated as more active, and they were more exploratory during the novel object test, which aligns with wild young rhinos being described as active and playful [10], as they are still testing their motor skills and learning new behaviors related to socialization and reproduction [23,50]. Thus, these observations could explain why ex situ males of both species and young white rhinos are more active than their counterparts.

Institution was related to how keepers rated their white rhinos as anthrophilic/-phobic (PC1) and reactive/stoic (PC4). The institution influence could be due to the unique perceptions of these traits by keepers within facilities [51], as well as differences in training style, and/or variation in staff-rhino interaction frequency and duration [52] among rhino-holding institutions.

Behavioral expression during the novel object test varied by enclosure type, which was chosen by the rhino care staff. Black rhinos expressed more olfactory behaviors with the basket in indoor, larger enclosures. It is possible that individuals may feel more comfortable and confident indoors where the environment is more controlled compared to outdoor enclosures where public access and noise levels are commonly higher, which are known to increase indicators of stress/anxiety in African rhinos [53–55]. As noted in other novel object test studies, in large enclosures, rhinos may have more sense of control and freedom to choose whether to interact with the basket than in small enclosures where they may not have the ability to avoid it [7,56]. This finding suggests that providing rhinos as much choice and control as possible could promote positive mental states by giving exploratory rhinos opportunities to investigate stimuli and avoidant rhinos opportunities to distance themselves from stimuli.

Behaviors in both assessments had more associations with demographic and environmental variables in white rhinos than in black rhinos. In a study comparing closely related gerbil species, Mongolian gerbils (*Meriones unguiculatus*) which are social species, exhibited higher behavioral plasticity by adjusting their behavior to different conditions than the midday gerbils (*Meriones meridianus*) which are known to be more solitary [57]. This difference between closely related species is consistent with results of the present study; the behavior of the white rhino, generally considered as the more social African rhino species [10], appeared to be more adaptable to different environmental conditions compared to black rhinos. However, it is important to note that the ability to adapt to changing conditions can potentially vary by individual, with some individuals having higher flexibility than others.

### Study design

Multiple factors need to be considered when interpreting results from temperament assessments. For instance, the novel object used could have affected the level of individual responses. In the present study, black rhinos rated as more fearful took less time to approach the basket. However, Carlstead et al. (1999) saw that black rhinos that took longer to touch a novel traffic cone were more fearful and avoidant [4]. Thus, black rhinos may perceive a 71 cm traffic cone [4] as riskier than the rope basket (34.8 cm x 24.9 cm x 20.0 cm) used in the present study. Horses, a close relative of rhinos, react differently to novel objects according to the shape and size [58]. Thus, the characteristics of the chosen novel object could affect how rhinos react to it even in closely related species. Therefore, when selecting a novel object, rhino caretakers need to consider an object that is risky enough to assess boldness/fearfulness and novel enough to assess exploration/avoidance traits. Additional considerations should include social tendencies of the species when isolating them during the novel object test, which could influence perceived risk.

Temperament surveys can be inconsistent between studies because they vary with trait definitions and their interpretations by raters. Further, high variability among keepers in the ratings within different behaviors, as seen in the present study, can occur [4,18,59,60]. Keeper experiences can contribute to the variability. For instance, animals in human care may display more species appropriate behaviors when keepers are present, particularly those related to activity [51]. Other factors may influence reliability, including the amount of time and frequency of keeper interactions with their animals, the bond between rhinos and each individual keeper, the number of individuals completing the surveys and size of the Likert scale used [4,18,59–61]. Therefore, cautious interpretation is advised for temperament surveys with low IRR, as in the present study. By calculating a median score, it is possible that extremes in rhino temperaments have been minimized, which also might dilute relationships that otherwise could possibly be detected between temperament surveys and behavioral performance on the novel object tests. This might have contributed to the absence of correlations between the two assessments following correction for a false discovery rate. Future studies should aim to improve IRR by having more keepers complete the surveys and collecting possible explanatory information for low reliability about training and interaction frequencies. In cases when training and keeper interactions are infrequent, a novel object test might be a better way to assess bold and exploratory tendencies.

Furthermore, novel object tests could be streamlined in future tests by excluding behaviors that rarely occurred or showed no correlation to survey rated behaviors. In both species, these behaviors included charging and head flinging toward the basket, and snort, snarl and whine vocalizations. In white rhinos, behaviors that showed no correlation to survey rated behaviors included time active, time to approach the basket, advancing directly and attacking the basket, and olfactory behaviors to other than the basket. Excluding these behaviors for the novel object test could improve the feasibility of this tool to make it more accessible to rhino caretakers.

### Implications for management and wellbeing

Knowledge of an animal’s temperament [4,20,21] can help provide management recommendations related to their environment, reproduction, housing compatibility, ability to cope with stress, health, and even translocation events [4,19,21,62–64]. The Five Domains Model of Wellbeing suggests that individuals express their mental states through behavioral interactions with their environment, conspecifics, and people [7]. Thus, rhino temperament could be a tool to understand African rhino mental state. Furthermore, temperament assessments provide a thorough profile of each rhino by evaluating multiple aspects of rhino personality, including traits related to exploration, boldness, anthrophilia, sociability, and activity. Managers can obtain these profiles via surveys and/or novel object tests to provide individualized care, according to each rhino temperament, that best allows rhinos to express natural behaviors and enhance positive effects of wellbeing.

Positive effects of wellbeing [6] are promoted through appropriate activity levels, exploration, social engagement (social species), and calmness or boldness [6,7]. The presentation of options and novel experiences is known to promote exploration [6,8,21,65] and activity [66], while offering choices also allows a sense of control [56,67], which can promote boldness [67] and social engagement [68,69]. For example, provision of novel objects to chimpanzees (*Pan troglodytes*) promoted exploration and activity, and reduced stereotypies [66], whereas offering indoor/outdoor access choice promoted affiliative behaviors with conspecifics and activity [69]. The key finding of the present study is that rhino temperaments are multi-faceted for each rhino and diverse among rhinos. As a result, no single management tactic will meet the mental needs of every rhino. Animal managers will need to be attentive to the type of temperament exhibited by their rhino(s) when developing enrichment, training, and husbandry plans. For example, managers possibly would develop a program that is more gradual and uses a higher reward frequency for training voluntary blood draw procedures with a more anthrophobic rhino compared to a more anthrophilic rhino. Furthermore, animal managers will need to assess the responses of their rhinos to the socio-environmental conditions that are provided to ensure the desired effect is achieved. For instance, whether provision of puzzle feeders, intended to occupy more exploratory rhinos, achieves the goal of increasing active and exploratory behavior should be measured through behavioral observations. This evaluation step utilizing measurable indicators, such as behavioral frequencies and durations, is a critical component of zoo animal welfare assessments [70].

Zoos housing multiple rhinos have the added challenge of promoting positive wellbeing in a way that simultaneously accommodates the expression of the different temperaments of all their rhinos. When those rhinos share the same space, one option for managers might be to provide large, complex environments. Sufficiently large enclosures promote choice and control by providing 1) free movement for active rhinos with resting areas for inactive rhinos, 2) access to multiple hiding spots and safe areas that limit external stimuli for fearful and avoidant rhinos, as well as multiple exit routes away from high-stimulus areas, and 3) ample space for diverse placement of enrichment items or novel situations (e.g., a fresh dirt mound) for exploratory and bold rhinos.

Rhino-holding facilities often have some limitations in terms of enclosure location and space, as well as opportunities for conspecific and human interactions. In such cases, use of temperament assessments to identify individuals that can best thrive in the available environment prior to their relocation to that facility might be advantageous. For instance, a fearful rhino that gets triggered by high human disturbance would benefit from an enclosure located in a quieter area or possibly a more safari-based zoological park. The present study is the first step in a larger study focused on how the expression of temperament for black and white rhinos relate to the other aspects of wellbeing, including: activity budgets, reproduction, nutrition and health. Consideration of a rhino’s normal activity level, exploratory, bold, reactive, anthrophilic, and sociable traits with conspecifics allows tailoring expectations for daily behaviors, interactions with enrichment and people. Optimizing individual care in these ways could enhance positive mental states and overall wellbeing. Future studies comparing temperament, activity, and other species-specific behaviors can provide insights into how different temperaments manage daily activities (e.g., social, exploratory, among other behaviors).

## Conclusion

1 Rhino temperament is complex; multiple dimensions obtained from the PCA provided evidence of multifaceted individual rhino temperaments.
2 Temperament surveys completed by keepers and novel object tests conducted by people not familiar with the rhino can provide reliable assessments of temperament.
3 The relationship of demographic and environmental factors to temperament expression demonstrates the need to consider those variables when interpreting rhino temperament data.
4 Study design (i.e., novel object used and definition of behaviors) needs to be considered when interpreting the results of both assessments.

## Supporting information

S1 File Novel_Obj_final. (CSV)

S2 File Temp_Survey_final. (CSV)

S1 Table Black (n = 50) and white rhino (n = 125) information from study population. Rhinos and their holding institutions were provided with unique identifications (Rhino ID and Institution ID respectively). Age of the rhino at the start of the study (in 2023), sex and the studies they participated in are shown as well. An asterisk (*) by the Rhino ID indicates the rhino was tested with a calf present.
(PDF)

S2 Table Pearson’s correlation for temperament survey ratings for all black (n = 49) and white rhinos (n = 125). Variables where the coefficient was ≥ |0.60| were either removed or consolidated. BR: sleep was correlated with active, thus sleep was removed and active retained. Familiar and stranger affiliative, familiar and stranger aggressive, and familiar and stranger fearful, were consolidated into single behaviors named “affiliative”, “aggressive”, and “fearful”, respectively. WR: sleep was correlated with active, and calm with stranger fearful, thus sleep and calm were removed and active and stranger fearful retained. Familiar aggressive and stranger aggressive were consolidated into a single behavior named “aggressive”. Pearson’s correlation coefficients ≥ |0.60| are shown in bold.
(PDF)

S3 Table Pearson’s correlation for temperament survey ratings for socially-housed black (n = 16) and white rhinos (n = 108). Variables where the coefficient was ≥ |0.60| were either removed or consolidated. BR: preferred object, assertive, and social were all highly correlated with each other, novel object and/or chemosensory; thus, they were removed. Conspecific aggressive was consolidated with food aggressive into a single behavior named “conspecific aggressive”. WR: preferred object was highly correlated with novel object, conspecific affiliative with social, and conspecific aggressive with food aggressive and assertive. Thus, preferred object, social, and assertive were removed. Conspecific aggressive and food aggressive were consolidated into a single behavior named “conspecific aggressive”. Correlation coefficients ≥ |0.60| are shown in bold.
(PDF)

S4 Table Spearman’s correlation for novel object test behaviors using frequencies and proportions in black (n = 32) and white rhinos (n = 66). Spearman’s correlation for novel object test behaviors using frequencies and proportions in black (n = 32) and white rhinos (n = 66). Variables where the coefficient was ≥ |0.60| were removed. BR: unstimulated and inactive behaviors were removed due to their respective correlations with stimulated and active. WR: Contact interaction, unstimulated, and gate wait behaviors were removed because they were all correlated with inactive. Spearman’s rho values ≥ |0.60| are shown in bold.
(PDF)
