# Supplementary material for: Temperament assessment of black (Diceros bicornis) and white (Ceratotherium simum) rhinos using keeper temperament surveys and novel object tests
Source: PLoS One. 2026 Sep 16;21(9):e0356484. doi: 10.1371/journal.pone.0356484 (PMC13581018; doi:10.1371/journal.pone.0356484)
Supplement: Supplementary file 1 [file pone.0356484.s003.pdf]

S1 Table. Black and white rhino information from study population. Rhinos and their holding institutions were provided with unique identifications (Rhino ID and Institution ID respectively). Age of the rhino at the start of the study (in 2023), sex and the studies they participated in are shown as well. An asterisk (*) by the Rhino ID indicates the rhino was tested with a calf present.

Rhino ID	Institution ID	Age	Sex	Novel Object	Temperament Survey
**Individually-housed Black Rhino**					
14662147	Inst-6	12	Male	No	Yes
21286963	Inst-10	33	Female	No	Yes
14662231	Inst-8	32	Male	Yes	Yes
19990823	Inst-24	31	Male	Yes	Yes
20285051	Inst-19	32	Female	No	Yes
14662249	Inst-10	30	Male	No	Yes
14662255	Inst-16	29	Male	Yes	Yes
14662288	Inst-21	26	Male	Yes	Yes
14662309	Inst-21	25	Female	Yes	Yes
21385657	Inst-15	29	Female	Yes	Yes
17244422	Inst-19	23	Female	No	Yes
21385660	Inst-15	22	Male	Yes	Yes
19990829	Inst-18	21	Male	Yes	Yes
19317336	Inst-12	21	Male	Yes	Yes
14662333	Inst-19	21	Male	No	Yes
19990805	Inst-18	20	Male	Yes	Yes
21385654	Inst-15	19	Female	Yes	Yes
14662342	Inst-24	18	Female	Yes	Yes
14662345	Inst-14	18	Male	Yes	Yes
14662348	Inst-40	14	Male	Yes	Yes
19030319	Inst-40	15	Female	Yes	Yes
19990817	Inst-24	15	Female	Yes	Yes
24428346	Inst-49	15	Male	Yes	Yes
19030340	Inst-8	11	Female	Yes	Yes
19990820	Inst-24	11	Male	Yes	Yes
19990814	Inst-21	26	Male	Yes	Yes
22939949	Inst-5	5	Male	No	Yes
24403824	Inst-5	5	Female	No	Yes
21190285	Inst-9	4	Female	Yes	Yes
22939850	Inst-51	4	Female	Yes	Yes
24688568	Inst-25	3	Male	No	Yes
24215274	Inst-51	3	Male	Yes	Yes
14662351*	Inst-14	12	Female	Yes	Yes
19298676	Inst-2	18	Male	Yes	No
**Socially-housed Black Rhino**					
14662153	Inst-55	12	Male	No	Yes
14662156	Inst-55	12	Female	No	Yes
19030328	Inst-6	5	Female	Yes	Yes
19030325	Inst-6	3	Female	Yes	Yes
17244386	Inst-19	26	Female	No	Yes
19024655	Inst-50	24	Female	Yes	Yes
20285048	Inst-19	22	Male	No	Yes

24428157	Inst-41	22	Female	Yes	Yes
24428154	Inst-41	19	Male	Yes	Yes
24428337	Inst-46	19	Male	No	Yes
24428340	Inst-46	18	Female	No	Yes
24422649	Inst-25	17	Female	Yes	Yes
20563568	Inst-27	16	Female	No	Yes
14662354	Inst-6	11	Female	Yes	Yes
23216989	Inst-36	10	Female	No	Yes
23215384	Inst-36	9	Male	No	Yes
**Individually-housed White Rhino**					
14662486	Inst-52	32	Male	Yes	Yes
14662492	Inst-55	31	Male	No	Yes
24428151	Inst-38	20	Male	No	Yes
15458923	Inst-42	20	Male	No	Yes
24428166	Inst-44	19	Male	Yes	Yes
14662534	Inst-19	18	Male	Yes	Yes
24428313	Inst-44	18	Male	Yes	Yes
24429882	Inst-56	11	Male	No	Yes
19230309	Inst-39	6	Male	Yes	Yes
24422010	Inst-61	7	Male	No	Yes
24424419	Inst-32	3	Male	Yes	Yes
15458941	Inst-42	49	Female	No	Yes
19298571	Inst-28	7	Male	No	Yes
19947173	Inst-26	55	Male	Yes	Yes
24403803	Inst-19	44	Female	No	Yes
19298643	Inst-28	38	Female	No	Yes
14662651	Inst-1	36	Male	Yes	Yes
**Socially-housed White Rhino**					
19990826*	Inst-26	23	Female	Yes	Yes
14662504*	Inst-18	26	Female	Yes	Yes
14662510*	Inst-10	28	Female	Yes	Yes
24421821*	Inst-20	17	Female	Yes	Yes
14662558*	Inst-52	13	Female	Yes	Yes
19990808*	Inst-18	12	Female	Yes	Yes
14662576*	Inst-52	11	Female	Yes	Yes
14662582*	Inst-52	10	Female	Yes	Yes
14662585*	Inst-54	10	Female	Yes	Yes
14662588*	Inst-52	9	Female	Yes	Yes
19990835*	Inst-18	7	Female	Yes	Yes
17283611	Inst-4	30	Female	No	Yes
24404031	Inst-59	31	Male	No	Yes
23877385	Inst-23	28	Female	No	Yes
24404058	Inst-59	32	Female	No	Yes
22201313	Inst-47	32	Male	Yes	Yes
14662471	Inst-30	27	Female	Yes	Yes
19990832	Inst-18	27	Male	Yes	Yes
19940654	Inst-26	26	Female	No	Yes
14662477	Inst-20	25	Male	Yes	Yes
21370591	Inst-26	25	Female	No	Yes
23728998	Inst-1	28	Male	Yes	Yes
22187249	Inst-1	27	Female	Yes	Yes

22187234	Inst-1	28	Female	Yes	Yes
22187246	Inst-1	27	Female	Yes	Yes
24424968	Inst-35	24	Male	No	Yes
14662489	Inst-4	31	Female	No	Yes
21639615	Inst-33	30	Female	No	Yes
21639468	Inst-33	34	Female	No	Yes
18673175	Inst-33	25	Female	No	Yes
20563577	Inst-3	37	Female	Yes	Yes
19990811	Inst-18	23	Female	Yes	Yes
24403821	Inst-4	24	Male	No	Yes
24429936	Inst-56	34	Male	No	Yes
24424971	Inst-35	36	Female	No	Yes
24403812	Inst-19	23	Female	No	Yes
19030334	Inst-17	22	Male	Yes	Yes
23031911	Inst-7	22	Female	Yes	Yes
16952462	Inst-4	22	Female	No	Yes
19030337	Inst-17	21	Male	Yes	Yes
14662498	Inst-45	26	Male	Yes	Yes
14662501	Inst-22	27	Female	Yes	Yes
14662507	Inst-10	28	Male	Yes	Yes
14662513	Inst-10	29	Female	Yes	Yes
24403896	Inst-7	21	Female	No	Yes
19990847	Inst-26	21	Female	No	Yes
21260167	Inst-7	20	Male	Yes	Yes
14662528	Inst-57	19	Male	Yes	Yes
19073289	Inst-43	20	Male	Yes	Yes
23217358	Inst-58	19	Male	No	Yes
23217481	Inst-58	19	Female	No	Yes
19548899	Inst-45	19	Female	Yes	Yes
23217421	Inst-58	19	Female	No	Yes
23217244	Inst-58	19	Female	No	Yes
14662537	Inst-52	18	Female	Yes	Yes
14662540	Inst-10	18	Female	Yes	Yes
21850740	Inst-37	17	Male	Yes	Yes
18673181	Inst-33	17	Female	No	Yes
21850731	Inst-37	17	Male	Yes	Yes
24428361	Inst-53	16	Male	Yes	Yes
19940438	Inst-26	14	Female	No	Yes
24428367	Inst-53	12	Female	Yes	Yes
24428382	Inst-53	13	Female	Yes	Yes
24424419	Inst-30	12	Male	Yes	Yes
24212772	Inst-54	17	Male	Yes	Yes
19939259	Inst-26	11	Female	No	Yes
14662570	Inst-52	12	Female	Yes	Yes
24403800	Inst-19	11	Female	No	Yes
17335112	Inst-43	12	Female	Yes	Yes
19990838	Inst-26	10	Male	Yes	Yes
20563574	Inst-3	10	Male	Yes	Yes
20553968	Inst-26	9	Female	No	Yes
14662579	Inst-52	8	Female	Yes	Yes
24403797	Inst-19	9	Male	No	Yes

14662591	Inst-54	9	Female	Yes	Yes
19030310	Inst-55	10	Female	No	Yes
22201325	Inst-47	7	Female	Yes	Yes
22201316	Inst-47	7	Female	Yes	Yes
23877388	Inst-23	6	Female	No	Yes
19030346	Inst-29	6	Male	Yes	Yes
19030349	Inst-29	6	Male	Yes	Yes
22742480	Inst-26	7	Female	Yes	Yes
19030316	Inst-55	11	Female	No	Yes
19030313	Inst-55	11	Female	No	Yes
24424725	Inst-31	7	Female	No	Yes
24424731	Inst-31	7	Female	No	Yes
24403809	Inst-19	5	Female	No	Yes
21503070	Inst-33	4	Female	No	Yes
24424800	Inst-33	4	Female	No	Yes
21286957	Inst-10	7	Female	Yes	Yes
21370588	Inst-26	3	Male	No	Yes
23215720	Inst-58	2	Female	No	Yes
19990844	Inst-26	3	Female	No	Yes
27596603	Inst-18	2	Male	No	Yes
21639474	Inst-33	2	Female	No	Yes
24429951	Inst-56	7	Female	No	Yes
24429918	Inst-56	7	Female	No	Yes
24429933	Inst-56	7	Female	No	Yes
18673169	Inst-33	2	Female	No	Yes
17335124	Inst-43	6	Female	Yes	Yes
14662600	Inst-62	46	Male	No	Yes
23877355	Inst-23	54	Male	No	Yes
14662624	Inst-22	43	Female	Yes	Yes
24428385	Inst-62	43	Female	No	Yes
19030331	Inst-33	54	Female	No	Yes
23577495	Inst-57	43	Female	Yes	Yes
19990841	Inst-26	42	Female	Yes	Yes
14662654	Inst-22	34	Male	Yes	Yes
