# Supplementary material for: Temperament assessment of black (Diceros bicornis) and white (Ceratotherium simum) rhinos using keeper temperament surveys and novel object tests
Source: PLoS One. 2026 Sep 16;21(9):e0356484. doi: 10.1371/journal.pone.0356484 (PMC13581018; doi:10.1371/journal.pone.0356484)
Supplement: Supplementary file 2 [file pone.0356484.s004.pdf]

S2 Table. Pearson's correlation for temperament survey ratings for all black (n=49) and white rhinos (n=125). Variables where the coefficient was ≥ |0.60| were either removed or consolidated. **BR:** sleep was correlated with active, thus sleep was removed and active retained. Familiar and stranger affiliative, familiar and stranger aggressive, and familiar and stranger fearful, were consolidated into single behaviors named "affiliative", "aggressive", and "fearful", respectively. **WR:** sleep was correlated with active, and calm with stranger fearful, thus sleep and calm were removed and active and stranger fearful retained. Familiar aggressive and stranger aggressive were consolidated into a single behavior named "aggressive". Pearson's correlation coefficients ≥ |0.60| are shown in bold.

	Investigative	Active	Calm	Sleep	Stereotypies	Familiar affiliative	Familiar aggressive	Familiar fearful	Responsive	Stranger affiliative	Stranger aggressive	Stranger fearful
**Black Rhino**												
**Investigative**	-											
**Active**	0.10	-										
**Calm**	0.51	-0.14	-									
**Sleep**	-0.13	**-0.64**	0.18	-								
**Stereotypies**	0.03	0.30	-0.06	-0.18	-							
**Familiar affiliative**	0.52	-0.14	0.50	0.04	-0.28	-						
**Familiar aggressive**	-0.23	0.21	-0.48	-0.12	0.40	-0.49	-					
**Familiar fearful**	-0.38	-0.09	-0.54	-0.05	0.18	**-0.60**	0.44	-				
**Responsive**	0.44	-0.15	0.34	0.22	-0.06	**0.77**	-0.42	-0.45	-			
**Stranger affiliative**	0.22	0.08	0.58	-0.06	-0.11	**0.60**	-0.50	**-0.68**	0.48	-		
**Stranger aggressive**	-0.02	0.00	-0.47	0.14	0.26	-0.38	**0.68**	0.36	-0.21	**-0.74**	-	
**Stranger fearful**	-0.16	-0.17	**-0.60**	-0.07	-0.08	-0.24	0.23	**0.67**	-0.23	**-0.80**	0.44	-
**White Rhino**												
**Investigative**	-											
**Active**	0.04	-										
**Calm**	0.30	-0.18	-									
**Sleep**	-0.13	**-0.63**	0.11	-								
**Stereotypies**	0.02	-0.11	-0.17	0.22	-							
**Familiar affiliative**	0.43	0.02	0.15	-0.02	-0.26	-						
**Familiar aggressive**	-0.22	0.00	-0.21	0.14	0.47	**-0.73**	-					
**Familiar fearful**	-0.25	0.03	-0.33	0.00	0.42	-0.37	0.28	-				
**Responsive**	0.21	0.00	0.03	0.18	-0.07	0.36	-0.14	-0.19	-			
**Stranger affiliative**	0.48	-0.10	0.35	0.02	-0.24	0.59	-0.48	-0.42	0.42	-		
**Stranger aggressive**	-0.18	0.07	-0.26	0.09	0.45	-0.55	**0.79**	0.29	-0.11	-0.54	-	
**Stranger fearful**	-0.39	0.08	**-0.66**	-0.14	0.26	-0.19	0.12	0.56	-0.16	-0.52	0.25	-
