# Supplementary material for: Temperament assessment of black (Diceros bicornis) and white (Ceratotherium simum) rhinos using keeper temperament surveys and novel object tests
Source: PLoS One. 2026 Sep 16;21(9):e0356484. doi: 10.1371/journal.pone.0356484 (PMC13581018; doi:10.1371/journal.pone.0356484)
Supplement: Supplementary file 3 [file pone.0356484.s005.pdf]

S3 Table. Pearson's correlation for temperament survey ratings for socially-housed black (n=16) and white rhinos (n=108). Variables where the coefficient was ≥ |0.60| were either removed or consolidated. **BR:** preferred object, assertive, and social were all highly correlated with each other, novel object and/or chemosensory; thus, they were removed. Conspecific aggressive was consolidated with food aggressive into a single behavior named "conspecific aggressive". **WR:** preferred object was highly correlated with novel object, conspecific affiliative with social, and conspecific aggressive with food aggressive and assertive. Thus, preferred object, social, and assertive were removed. Conspecific aggressive and food aggressive were consolidated into a single behavior named "conspecific aggressive". Correlation coefficients ≥ |0.60| are shown in bold.

	Preferred object	Novel object	Assertive	Chemosensory	Consp. affiliative	Consp. aggressive	Consp. fearful	Social	Vocal	Food aggressive
**Black Rhino**										
**Preferred object**	-									
**Novel object**	**0.73**	-								
**Assertive**	**0.69**	**0.65**	-							
**Chemosensory**	**-0.65**	-0.22	-0.58	-						
**Consp. affiliative**	-0.23	-0.11	**-0.77**	0.56	-					
**Consp. aggressive**	0.19	0.08	0.10	-0.58	-0.32	-				
**Consp. fearful**	-0.54	-0.38	**-0.78**	0.36	0.51	0.09	-			
**Social**	-0.22	-0.43	**-0.66**	0.18	0.39	0.35	**0.65**	-		
**Vocal**	0.27	0.13	-0.34	-0.32	0.41	0.54	0.58	0.59	-	
**Food aggressive**	0.47	0.32	0.11	**-0.71**	0.01	**0.62**	0.23	0.10	**0.82**	-
**White Rhino**										
**Preferred object**	-									
**Novel object**	**0.86**	-								
**Assertive**	0.32	0.41	-							
**Chemosensory**	0.00	-0.02	-0.39	-						
**Consp. affiliative**	-0.02	0.12	-0.08	0.15	-					
**Consp. aggressive**	0.40	0.40	**0.60**	-0.31	-0.28	-				
**Consp. fearful**	-0.04	-0.02	-0.52	0.39	-0.05	-0.07	-			
**Social**	-0.10	-0.11	-0.15	-0.11	**0.68**	-0.28	-0.10	-		
**Vocal**	0.30	0.33	0.30	-0.12	0.18	0.29	-0.03	0.26	-	
**Food aggressive**	0.31	0.39	**0.65**	-0.35	-0.14	**0.69**	-0.29	-0.09	0.32	-
