# Supplementary material for: Temperament assessment of black (Diceros bicornis) and white (Ceratotherium simum) rhinos using keeper temperament surveys and novel object tests
Source: PLoS One. 2026 Sep 16;21(9):e0356484. doi: 10.1371/journal.pone.0356484 (PMC13581018; doi:10.1371/journal.pone.0356484)
Supplement: Supplementary file 4 [file pone.0356484.s006.pdf]

S4 Table. Spearman's correlation for novel object test behaviors using frequencies and proportions in black (n=32) and white rhinos (n=66). Spearman's correlation for novel object test behaviors using frequencies and proportions in black (n=32) and white rhinos (n=66). Variables where the coefficient was ≥ |0.60| were removed. **BR:** unstimulated and inactive behaviors were removed due to their respective correlations with stimulated and active. **WR:** Contact interaction, unstimulated, and gate wait behaviors were removed because they were all correlated with inactive. Spearman's rho values ≥ |0.60| are shown in bold.

	Retreat	Olfactory w/object	Olfactory w/other	Advance direct	Contact interact	Near	Stimulated	Unstimulated	Active	Inactive	Gate wait	Latency approach	Pace
**Black Rhino**													
**Retreat**	-												
**Olfactory w/object**	-0.07	-											
**Olfactory w/other**	-0.12	0.34	-										
**Advance direct**	0.31	0.17	0.58	-									
**Contact interaction**	-0.14	0.41	0.21	0.14	-								
**Near**	-0.35	0.09	-0.03	-0.32	0.51	-							
**Stimulated**	0.49	0.24	0.19	0.51	-0.07	-0.43	-						
**Unstimulated**	-0.46	-0.14	-0.22	-0.51	0.23	0.43	**-0.74**	-					
**Active**	0.23	0.18	0.37	0.57	-0.02	-0.36	0.32	-0.59	-				
**Inactive**	-0.28	0.10	-0.24	-0.52	0.09	0.29	-0.23	0.63	**-0.89**	-			
**Gate wait**	0.42	-0.04	0.21	0.52	0.22	-0.03	0.30	-0.28	0.26	-0.42	-	-	
**Latency approach**	-0.07	0.35	0.01	0.07	0.01	-0.14	0.06	0.16	-0.18	0.32	-0.05		
**Pace**	0.21	-0.25	0.09	0.20	-0.34	-0.16	-0.01	-0.55	0.55	**-0.72**	0.07	-0.36	-
**White Rhino**													
	**Pant**	**Olfactory w/object**	**Olfactory w/other**	**Advance direct**	**Contact interact**	**Attack**	**Near**	**Stimulated**	**Unstimulated**	**Active**	**Inactive**	**Gate wait**	**Latency approach**
**Pant**	-												
**Olfactory w/object**	0.02	-											
**Olfactory w/other**	0.34	0.00	-										
**Advance direct**	0.07	0.38	0.22	-									
**Contact interaction**	-0.10	**0.64**	0.02	0.41	-								
**Attack**	0.32	0.41	0.06	0.27	0.34	-							
**Near**	-0.13	0.35	-0.03	0.16	0.41	**0.09**	-						
**Stimulated**	0.53	0.27	0.30	0.02	0.14	0.45	-0.08	-					
**Unstimulated**	-0.53	-0.27	-0.30	-0.02	-0.14	-0.45	0.08	**-1.00**	-				
**Active**	0.26	0.20	0.50	0.34	0.10	0.30	-0.06	0.18	-0.17	-			
**Inactive**	-0.37	-0.03	-0.26	-0.25	0.02	-0.29	-0.01	-0.16	0.16	-0.49	-		
**Gate wait**	0.56	0.03	0.14	-0.01	0.01	0.31	0.02	0.54	-0.54	0.17	**-0.62**	-	
**Latency approach**	-0.05	0.00	0.07	0.17	-0.13	-0.14	0.00	-0.11	0.11	-0.03	-0.07	-0.02	-
